# The Formidable yet Unresolved Interplay between Endometriosis and Obesity

**DOI:** 10.1155/2021/6653677

**Published:** 2021-04-20

**Authors:** Athanasios Pantelis, Nikolaos Machairiotis, Dimitris P. Lapatsanis

**Affiliations:** ^1^Surgeon, 4th Department of Surgery, Evaggelismos General Hospital of Athens, Ipsilantou 45, Athens 106 76, Greece; ^2^Fellow in Endometriosis and Minimal Access Surgery, Northwick Park, Central Middlesex and Ealing Hospitals, London North West University Heathcare, NHS Trust, London, UK

## Abstract

Obesity and endometriosis are two very common entities, yet there is uncertainty on their exact relationship. Observational studies have repeatedly shown an inverse correlation between endometriosis and a low body mass index (BMI). However, obesity does not protect against endometriosis and on the contrary an increased BMI may lead to more severe forms of the disease. Besides, BMI is not accurate in all cases of obesity. Consequently, other anthropometric and phenomic traits have been studied, including body adiposity content, as well as the effect of BMI early in life on the manifestation of endometriosis in adulthood. Some studies have shown that the phenotypic inverse correlation between the two entities has a genetic background; however, others have indicated that certain polymorphisms are linked with endometriosis in females with increased BMI. The advent of metabolic bariatric surgery and pertinent research have led to the emergence of biomolecules that may be pivotal in understanding the pathophysiological interaction of the two entities, especially in the context of angiogenesis and inflammation. Future research should focus on three objectives: detection and interpretation of obesity-related biomarkers in experimental models with endometriosis; integration of endometriosis-related queries into bariatric registries; and multidisciplinary approach and collaboration among specialists.

## 1. Introduction

Endometriosis is a disease with high prevalence among women of reproductive age, affecting approximately 10% of this population [1, 2]. These figures skyrocket when investigating specific subpopulations: the incidence of endometriosis ranges from 35–100% in symptomatic females who undergo laparoscopic visualization, whereas the respective estimate among infertile women is 5–50% [3]. Obesity, on the other hand, had affected approximately 650 million adults as of 2016, whereas almost 2 billion have been characterized as overweight, according to the World Health Organization (WHO) [4]. In other words, almost 40% of the world adult population is deemed as overweight and 13% as obese, justifying the characterization of obesity as a modern pandemic.

Over time, there has been accumulating evidence of an inverse correlation between endometriosis and body mass index [5–10]. This has been supported largely by observational data; subsequently, the establishment of causality is debatable. The interpretation could be purely mechanical: retrograde menstruation, a widely accepted initiator of endometriosis [3, 11], may be a more frequent phenomenon in nonobese subjects due to reduced intraabdominal pressures. Another explanation could entail behavioral factors, as the chronic pain that heralds endometriosis, along with accompanying gastrointestinal disturbances (nausea and diarrhea), may lead to loss of appetite and restriction of food intake [12]. In fact, the inverse correlation of endometriosis and body weight may seem counterintuitive, as both conditions are related to hyperestrogenemia and systemic inflammation [12]. In this context, emphasis should be placed on the metabolic status (i.e., diabetes, hypertension, dyslipidemia, and adiposity) of a woman rather than solely on their weight.

These data indicate that elucidating the actual connection between endometriosis and obesity is a challenging yet promising task. Indeed, the Global Consortium of Investigators in Endometriosis has deemed this issue of high priority and has recommended that “studies (should) be undertaken to investigate the relationship between phenotypic variables, including BMI and endometriosis” in their latest declaration [13]. The present review is organized in four sections: (i) evidence on the relation between BMI and endometriosis; (ii) anthropometric correlations beyond BMI; (iii) interconnection of the two entities on genetic, molecular, and pathophysiological levels; (iv) future insights and research proposals from a bariatric perspective.

## 2. Materials and Methods

The process for identifying pertinent literature entailed online search in three electronic databases, Pubmed, Embase, and Web of Science, for a period of three months (1^st^ May–31^st^ July 2020). All relevant studies in English language published after 2000 were considered for reviewing. Key search terms included terminology for (“obesity” OR “obese” OR “body mass index” OR “BMI” or “adiposity” OR “body size” OR “bariatric surgery” OR “metabolic surgery” OR “adiponectin” OR “chemerin” OR “ghrelin” OR “leptin”) AND (“endometriosis”). In addition to primary research, key references of the studies were retrieved, and seminal studies were also considered. In total, 62 full manuscripts were obtained, including clinical and experimental research papers, systemic reviews and meta-analyses, narrative reviews, editorials, and letters to the editor.

## 3. The Inverse Correlation between Endometriosis and BMI, Which More than Meets the Eye

The most widely accepted measure of obesity is body mass index (BMI). BMI is not ideal, as it does not take into consideration different components of excess weight (i.e., adipose tissue vs. muscular mass), different properties of adipose tissue by location (subcutaneous vs. visceral), gender influences or familial predisposition, and genetic factors [12, 14]. Nevertheless, the clinical application of BMI is widespread, thanks to its convenience of calculation without the need of sophisticated or invasive equipment and its utility for defining and grading obesity, as well as for assigning candidacy for bariatric operations and for comparisons between individuals, groups, and interventions [15]. For adults, the WHO defines overweight as BMI ≥25 Kg/m^2^ and obesity as BMI ≥30 Kg/m^2^ [4].

Since the seminal publications of Hemmings et al. in 2004 (prospective cohort) [16] and Ferrero et al. in 2005 (case-control) [6], there have been numerous reports underlining the inverse correlation between endometriosis and BMI. Among them, there are two that stand out due to methodology and large sample sizes. Shah et al. prospectively studied a cohort of 116,430 female nurses (Nurses' Health Study II-NHS II) for a 22-year period, which yielded 5,504 cases of laparoscopically-confirmed endometriosis [8]. Overweight women with endometriosis had a relative risk (RR) of 0.99 (95% CI 0.91–1.06), whereas there was a gradual lowering of this risk with increasing BMI (30–34.9: RR 0.94, 95% CI 0.85–1.05; 35–39.9: RR 0.96, 95% CI 0.83–1.11; >40 : 0.61, 95% CI 0.50–0.75). As shown, there was a statistically significant inverse prevalence of endometriosis in females with high-grade obesity. Inversely, the RR for endometriosis in underweight women (BMI <18.5) was 1.16 (95% CI 0.97–1.38). More recently, Saha et al. conducted a cross-sectional study that included 28,822 women, 1,228 with endometriosis and 27,594 controls [17]. Overweight women had an adjusted odds ratio (OR) for endometriosis of 1.49 (95% CI 0.93–2.37), whereas the respective figure for obese women was 1.36 (95% CI 0.82–2.26). The adjusted OR of underweight women for endometriosis was 1.30 (95% CI 0.82–2.22). Although these figures did not yield statistically significant results, it should be noted that there was no stratification of obesity into grades, as was the case with the study of Shah et al.

The abundance of relevant studies and simultaneously the need to clarify the situation are depicted on the fact that there are two high-quality meta-analyses on the association between BMI and the risk of endometriosis, one by Liu and Zhang [18] and one by Jenabi et al. [18, 19]. Interestingly, after the implementation of inclusion and exclusion criteria, the two meta-analyses shared only three studies, even though they have been published only two years apart. Except for the chronological discrepancies, the two meta-analyses also featured some methodological differences, summarized in Table 1. In brief, the meta-analysis of Liu and Zhang suggested that a higher BMI may be associated with lower risk of endometriosis, whereas Jenabi et al. found that underweight was a risk factor for endometriosis, while obesity did not seem to have a statistically significant protective effect (Table 1).

Three newer studies have attempted to shed light. Holdsworth-Carson et al. have retrospectively studied an Australian population of 509 women, 357 with laparoscopically proven endometriosis and 152 controls [20]. The former group comprised 16 (88.9%) underweight, 90 (70.9%) overweight, and 51 (52.6%) obese subjects, whereas the latter consisted of 2 (11.1%) underweight, 37 (29.1%) overweight, and 46 (47.4%) obese individuals (*p* < 0.001). Interestingly, this study also found that obesity was linked to increased severity of endometriosis, reduced frequency of stage I disease, and significantly higher revised American Fertility Society (rAFS) scores. On the contrary, the study of a Korean female population by Yun et al. with 134 histologically confirmed endometriosis cases and 282 ovarian teratoma controls did not reveal any association between BMI and severity of disease [21]. Endometriosis cases had a statistically significant lower mean BMI as compared to controls (BMI_cases_: 21.43 [19.59–23.61] vs. BMI_controls_: 22.19 [20.41–24.61], *p* 0.014). The third study was conducted on 250 Iranian women and found that 125 women with endometriosis in the case group had a mean BMI of 23.07 ± 3.06, which was significantly lower than the mean BMI of 32.26 ± 4.01 of the 125 controls (*p* 0.002) [10].

The evidence so far showcases that there is indeed an inverse correlation between endometriosis and BMI, but by no means, one may extrapolate that obesity plays a protective role against endometriosis or vice versa. Consequently, research should focus on different anthropometric measures, potential molecular and genetic connections, or even alternative methodological approaches to elucidate the complex relationship between obesity and endometriosis.

## 4. Beyond BMI, Anthropometric and Phenomic Parameters that Link Endometriosis and Obesity

As stressed previously, BMI has some inherent weaknesses as a surrogate measure of obesity. In the context of endometriosis, solely examining the role of BMI may act as a confounder. Firstly, BMI is not stable over a woman's lifetime. Most importantly, some women may weight within normal range but be metabolically abnormal due to maldistributed adiposity or excess body fat (i.e., >30% of total body weight), a condition recognized as normal weight obesity syndrome (NWOS) [12, 22, 23]. The estimated prevalence of NWOS is 24% and patients are characterized by normal weight, minimal physical activity, BMI in the range of 20–27 Kg/m^2^ with a fat mass of 2–10 Kg, and a predisposition to prediabetes or borderline dyslipidemia [24]. In these women, the impact of obesity on endometriosis cannot be measured by means of BMI; consequently, other parameters, such as lifestyle modifications and anthropometric measurements of adiposity, should be taken into account [10, 12].

Phenomics is a relatively novel field that involves the mapping of disease networks, in an attempt to establish connection between disease process through shared phenotypic or molecular pathways [25]. A pivotal concept of phenomics is the role of environmental exposures and genotype-environmental interactions. Given that, on the one hand, endometriosis has a poorly defined phenotype, and on the other hand, obesity has complex and multifactorial environmental influences; it may be challenging to eliminate these barriers. An interesting approach is the association of endometriosis with body size during childhood, puberty, and early adulthood, in an attempt to mitigate environmental influences that accumulate over time. Viganò et al. collected five early such studies, all of which demonstrated a consistent and strong inverse association between endometriosis and lean early life body size, even after stratification for age, birthweight, age at menarche, parity, and oral contraceptive use [25]. The biggest disadvantage of these studies was recall bias, as participants were retrospectively interviewed and asked to remember their weight at earlier ages. Most recently, Aarenstrup et al. included 171,447 girls from a national health records register with measured heights and weights [26]. Among 2,149 cases, endometriosis was inversely associated with childhood BMI (per *z*-score at age 7) with a hazard ratio (HR) of 0.92 (95% CI 0.88–0.96) and was positively associated with childhood height (HR 1.09, 95% CI 1.05–1.14), thus suggesting that a lean and tall phenotype in a girl correlates with the development of endometriosis later in life. These findings are in accordance with the NHS II, which found a strong inverse trend between BMI at age 18 and endometriosis risk (*p* ≤ 0.0006), even after adjusting for fertility, current BMI, or cycle regularity [8].

Another strategy is to study the association of anthropometric measures and body composition indicators other than BMI in order to overcome the intrinsic limitations of the latter. Backonjia et al. performed a series of such calculations in 473 females and found statistically significant relationships of endometriosis with weight, subscapular skinfold thickness, waist and hip circumferences, and total upper arm and upper arm muscle areas [27]. Even in this study, BMI had a relatively strong inverse association with endometriosis (adjusted OR 0.75, 95% CI 0.60–0.93), second only to upper arm muscle area (adjusted OR 0.74, 95% CI 0.65–0.98). Interestingly, this study failed to show any significant association between body fat distribution and endometriosis, thus challenging the concept of NWOS and its impact on this population. More recently, Byun et al. postulated that adiposity may be linked to endometriosis severity, as women with American Society of Reproductive Medicine (ASRM) stages I or IV endometriosis had lower values of adiposity indicators as compared to women with stage II or III disease [28]. Moreover, they correlated fat content with disease typology, because women with ovarian endometrioma and/or deep infiltrating endometriosis had low adiposity indicator values, in contrast to those with superficial endometriosis who had the highest values. Notably, all measurements had considerable overlapping: only three reached statistical significance for disease severity (weight, BMI, and midupper arm circumference) and one for disease typology (BMI).

## 5. Searching in the Genes, How Obesity and Endometriosis Interact at a Genetic Level

The consistently strong correlation between low BMI and endometriosis has directed research towards potential genetic associations. Goetz et al. studied the impact of endometriosis on hepatic gene expression on an animal model and found that four genes related to weight loss were upregulated, whereas two genes linked to obesity were suppressed [29]. Additionally, two key metabolic genes (*Lep* and *Pparg*) that are involved in the expression of the aforementioned ones were also upregulated. This study has suggested a genetic basis for the observed inverse correlation between endometriosis and BMI. In contradistinction, a genome-wide analysis revealed that a specific intergenic locus on 7p15.2 was associated with both endometriosis and fat distribution [30]. This unexpected finding prompted further investigation and retrieval of four additional genetic loci that have a statistically significant overrepresentation in both endometriosis and fat distribution. Similarly, Vilarinho Cardo et al. found that a specific polymorphism of an isoform of the cytochrome (CYP2C19*∗*2) had a positive correlation with endometriosis in females with increased BMI (range 30–40) [31]. These findings suggest that, despite the observation that women with endometriosis have a low BMI, obesity does not protect from the manifestation of endometriosis, in accordance with the meta-analysis of Jenabi et al. [19].

The biological function of microRNAs is a promising contemporary concept in the pathophysiology of endometriosis [32]. Zolbin et al. demonstrated that the upregulation (miR-342-3p) or inhibition (Let-7b) of microRNAs seen in endometriosis significantly alters the expression of adipocyte genes related to brown adipocyte differentiation (*Cebpa/b* and *Ppar-γ*), fat metabolism (*IL-*6, *HSL*), and glucose metabolism (*leptin* and *adipoq*) [33]. However, this relationship does not seem to be reciprocal: Holdsworth-Carlson found no significant impact of obesity on endometrial gene expression, even after stratification for BMI or severity of endometriosis [34]. In conclusion, evidence of a genetic crosstalk between endometriosis and obesity is emerging but remains elusive; more pioneer studies as the ones mentioned previously are necessary.

## 6. Metabolic Surgery, What It Is and How It Can Contribute to Endometriosis Research

According to the latest annual report of the International Federation for the Surgery of Obesity and Metabolic Disorders (IFSO^®^) global registry, 833,687 operations had been performed until 2019, thus confirming the pivotal role of bariatric surgery in the battle against obesity [35]. Obesity is accompanied by a constellation of metabolic irregularities, the most prominent of which is type 2 diabetes mellitus (T2DM). The superior efficacy and effectiveness of bariatric operations against T2DM and other metabolic comorbidities as compared to medical treatment have been shown in multiple high-quality studies and meta-analyses [36–40]. Consequently, there has recently been adopted a terminology shift from purely “bariatric” to “metabolic bariatric” surgery [41].

But there is more to it than simply a name change: clinical findings have been supported by pathophysiological insights, as new molecules and mechanisms have been discovered in an attempt to decipher how these operations work [42, 43]. Obesity, along with the events that follow MBS, are heralded by a characteristic endocrine and molecular milieu, the study of which has contributed to a paradigm shift regarding the function of the gastrointestinal tract and adipose tissue as powerful endocrine “organs” [44]. The expression of several among these molecules seems to be altered also in endometriosis. The role of four such molecules will be further analyzed herewith.

### 6.1. Ghrelin

Ghrelin is an orexigenic hormone that is secreted primarily by the gastric fundus, acts on the arcuate and solitary nuclei of the hypothalamus, and stimulates the release of growth hormone [45]. Ghrelin receptors have widespread distribution across body tissues [46], thus implicating a multifactorial role in various functions and processes [47]. Obesity is characterized by elevated ghrelin levels, decreased postprandial feedback inhibition, and increased sensitivity of its receptor across tissues [48]. Among bariatric operations, sleeve gastrectomy, which entails removal of the entire fundus, results in reduced ghrelin levels and consequently in reduced appetite [45, 48, 49]. The situation is more complicated for Roux-en-Y gastric bypass, the second most popular bariatric operation, as it is characterized by a short-term decrease in ghrelin levels, followed by an increase at 3 months postoperatively [50] and further decline at 12 months [51].

In 2009, Dziunycz et al. collected peritoneal fluid samples from 46 nonobese subjects and detected increased levels of ghrelin [52]. Ghrelin concentrations had a statistically significant correlation with the grade of endometriosis, as well as a strongly positive association with VEGF (*r*_*s*_ 0.625), a major angiogenetic mediator. Interestingly, ghrelin levels were not correlated with inflammatory cytokines classically associated with endometriosis, such as interleukin (IL-1*β*) 1*β*, IL-6, and tumor necrosis factor (TNF). Despite its limitations due to a relatively low sample size, this was the first study to suggest a role of ghrelin in the pathophysiology of endometriosis. A few years later, the same investigators studied the expression of ghrelin and the localization of its GHSR1a receptor in endometriotic tissue samples from 20 women [53]. They found high expression of both ghrelin and its receptor in endometrioid glandular epithelium, as well as in the endothelium of stromal blood vessels of the same tissue and in leukocytes isolated from the peritoneal fluid. The latter findings were suggestive of the involvement of ghrelin with two key processes in the pathophysiology of endometriosis, i.e., angiogenesis and inflammation.

The positive correlation of ghrelin with endometriosis is somewhat controversial, given the inverse correlation between BMI and endometriosis and the positive correlation between ghrelin and obesity. Indeed, Rathore et al. showed that the concentration of ghrelin in the peritoneal fluid of patients with endometriosis is significantly lower than that of controls (*p* 0.037) [54]. Additionally, they did not find any correlation between ghrelin and IL-6 or VEGF. However, this study has several limitations, the most serious being the lack of healthy controls, which prevents generalizability of the results.

### 6.2. Leptin

Leptin is a hormone normally secreted by the adipose tissue; hence the name of the relevant class of molecules is known as adipokines. The action of leptin is roughly opposite to that of ghrelin, but its exact role depends on the energy state of the body: in steady body weight, it reflects the content of body fat; in cases of weight excess, its increased concentrations reduce appetite through central nervous signaling, whereas in conditions of food restriction, its levels decline in order to meet increased energy demands [44]. Apart from its metabolic properties, leptin is also known as a proinflammatory and angiogenetic mediator [55–57]. Increased levels of leptin have been identified in serum and peritoneal fluid specimens from women with endometriosis for more than two decades [55, 58]. The role of leptin in the establishment and propagation of endometriotic lesions has also been shown in experimental models [59], but case-control analysis within the large cohort of NHS II failed to show statistically significant associations between endometriosis and leptin, even after adjustment for BMI (RR 1.2, 955 CI 0.7–2.0, *p* 0.72) [60]. More recently, Rathore et al. showed that women with endometriosis had higher peritoneal fluid concentrations of leptin as compared to nonendometriosis peers (*p* 0.040) [54]. They also found a significant correlation between leptin and IL-6, thus confirming the involvement of the former in inflammation. Again, the major disadvantage of the study was the absence of healthy individuals from the control group.

Other investigators have attempted to correlate leptin levels with disease typology. In a case-control study of 44 women with endometriosis and 42 healthy controls, Choi et al. showed that leptin had an expression rate of 100% in ovarian endometrioma tissue specimens versus 59.5% in normal endometrium (*p* < 0.001), independent of BMI or stage of disease [61]. Furthermore, Gonçalves et al. found that leptin concentration in peritoneal fluid did not differ between patients and control (*p* 0.18), and there was only borderline difference between those with ovarian implants and without them (*p* 0.048) [57]. On the contrary, serum leptin levels were significantly higher both in patients versus controls (*p* < 0.0001) and in those without ovarian implants versus those with implants (*p* 0.04).

### 6.3. Adiponectin

Adiponectin is another member of the adipokine family. Its levels are low in obesity and increase after weight loss, bariatric surgery, and especially RYGB [62, 63]. Initial studies showed that adiponectin secretion was suppressed in the context of endometriosis, both in the peritoneal fluid [64] and serum [65]. Nevertheless, the large NHS II study did not reveal any significant associations between endometriosis and adiponectin (RR 0.8, 95% CI 0.5–1.2, *p* 0.48) or leptin-to-adiponectin ratio (RR 0.8, 95% CI 0.4–1.4, *p* 0.14) after adjustment for BMI [60]. Similarly, the study of Choi et al. did not yield any difference in expression of adiponectin between ovarian endometrioma and normal endometrial tissue samples (31.8% vs. 42.9%, *p* 0.29) [61].

### 6.4. Chemerin

Chemerin holds a dual role as a chemoattractant and an adipokine, which renders it a pivotal player in bridging obesity and insulin resistance with inflammation [66, 67]. In a recent prospective study of 100 obese individuals, it has been shown that chemerin, along with leptin and ghrelin, has a sustainably low concentration one year following bariatric surgery [51]. Regarding endometriosis, Jin et al. have demonstrated that chemerin concentration was significantly elevated both in the peritoneal fluid and specimens of endometriotic tissue as compared to normal endometrial tissue [68]. What is more, chemerin concentration positively correlated with the levels of classical inflammatory mediators, such as IL-6 and TNF-*α* (*p* < 0.001).

### 6.5. Critical Appraisal on Biomolecules

Shared molecular pathways and biomarkers between endometriosis and obesity do not establish a causative relationship between the two entities. Consequently, further research is needed on experimental models of endometriosis and/or obesity with knockout properties or ablation of the molecule(s) under investigation. Regarding clinical trials, it is necessary to recruit large cohorts of patients and apply long-term surveillance periods to reach reliable conclusions, which is not always practical or cost-effective. Even in this case, the concentration of a biomarker in a given organic fluid or specimen needs cautious interpretation. For example, ghrelin is found in two isoforms, the most active of which (acylated) constitutes only 10% of the circulating molecule, a property that hardly any publications have taken into consideration [48]. Another area of uncertainty is standardization of methodology on all phases of testing (preanalytical, analytical, and postanalytical). Beyond demystifying mechanisms of disease, the utility of biomarkers is their potential use in clinical practice as diagnostic or predictive tools. To date, none of the recently discovered obesity-related molecules has proven its effectiveness in endometriosis [69, 70].

## 7. Conclusions and Future Insight

Given that BMI is the most widespread surrogate measure of obesity, most studies seem to converge on that there is an inverse correlation between endometriosis and obesity.  Table 2 summarizes relevant evidence from studies included in the current review. However, it must be emphasized that a high BMI does not preclude the development of endometriosis [19]. In the same context, the pivotal observation that obesity may be associated with severe forms of endometriosis should be investigated further [20].

Precise definition is key to understanding the interplay between endometriosis and obesity. We examined the potential pitfalls that may appear by deeming BMI synonymous with obesity and the need to develop indices that reflect an individual's metabolic imprint of adiposity more accurately but with equivalent usability [24]. Similar misunderstandings may derive by not specifying the stage and localization of endometriosis in clinical and experimental studies alike. Endometriosis is a heterogenous disease, as such different types may reflect different pathophysiologic pathways [3, 71, 72]. In this context, the adoption of a widely accepted classification system which allows for comparisons between different study populations is mandatory. In addition, the diagnostic challenge of endometriosis should preclude any diagnostic modality other than laparoscopyrch [2].

Considering the high prevalence of obesity and endometriosis, as well as the widespread bibliographic references on their correlation, it is remarkable that there is paucity of literature on the impact of metabolic bariatric surgery (MBS) on endometriosis. Did women who had undergone MBS and had significant and sustainable weight loss present with endometriosis? Does the “challenging diagnostic tetrad” of endometriosis make its diagnosis even more difficult in the post-MBS setting? What is the impact of MBS on women with endometriosis with regards to severity of symptoms or extent of disease? What alterations in inflammatory mediators and metabolic biomarkers are noted in these patients? To be addressed, these questions need large populations and long-term follow-up periods. A global registry such as the one endorsed by IFSO^®^ could serve as an excellent tool, by integrating input on endometriosis to the already existing databases. This approach also underlines that the key to understanding the underlying mechanisms is a multidisciplinary approach and collaboration between bariatric surgeons, gynecologists, endocrinologists, reproductive specialists, and other professionals.

## Figures and Tables

**Table 1:** Comparison between two meta-analyses examining the correlation between endometriosis and BMI.

Authors	Liu and Zhang [18]	Jenabi et al. [19]
Year of publication	2017	2019
Included studies	11	9
Cohort (%)	2 (18.2)	3 (33.3)
Case-control (%)	9 (81.8)	4 (44.4)
Cross-sectional (%)	0 (0.0)	2 (22.2)
Common studies	3	
Histologic diagnosis of endometriosis (%)	10 (90.9)	6 (66.7)
Heterogeneity-*I*^2^ (*p*)	86.9% (<0.001)	4.5% (0.40)
Correlation of endometriosis with		
Underweight	N/A	OR 1.41, 95% CI 1.16–1.66
Overweight	RR 0.97, 95% CI 0.91–1.05	OR 0.95, 95% CI 0.72–1.18
Obesity	RR 0.87, 95% CI 0.83–0.96	OR 0.88, 95% CI 0.54–1.21

**Table 2:** Summary of the included studies on the correlation between endometriosis and obesity. The studies that found no or positive correlation are in bold.

Type of evidence	Component	Seminal studies					Key findings
		First author	Year of publication	Ref. no.	Type of study	No. of subjects	
Clinical	BMI	Hemmings R.	2004	[16]	Case-control	2.777	Inverse correlation between endometriosis and BMI
		Ferrero S.	2005	[6]	Case-control	614	Inverse correlation between endometriosis and BMI
		Viganò P.	2012	[25]	Systemic review	187,340 (11 studies)	Modest inverse correlation between endometriosis and BMI
		Shah D. K.	2013	[8]	Prospective cohort	116.430	Inverse correlation between endometriosis and BMI
		**Shaha R.**	**2016**	17	**Cross-sectional**	**27.594**	**No correlation between endometriosis and BMI**
		Liu Y.	2017	[18]	Meta-analysis	128,944 (11 studies)	Inverse correlation between endometriosis and BMI
		Holdswoth-Carson S. J.	2018	[20]	Retrospective cohort	509	Inverse correlation between endometriosis and BMI
		Jenabi E.	2019	[19]	Meta-analysis	96,249 (9 studies)	Correlation between underweight and endometriosis but no (inverse) association between overweight and endometriosis
		Yun K. Y.	2020	[21]	Case-control	416	Inverse correlation between endometriosis and BMI
		Vaghar M. I.	2020	[10]	Questionnaire-based	250	Inverse correlation between endometriosis and BMI
	Phenomics	Viganò P.	2012	[25]	Systemic review	269,276 (5 studies)	Strong inverse correlation between endometriosis and early life body size
		Aarenstrup J.	2020	[26]	Review of national registry	171.447	Lean and tall girls are more often diagnosed with endometriosis in adult life
	Nonweight anthropometrics	Backonjia U.	2017	[27]	Cross-sectional	473	Inverse correlation between endometriosis and weight; subscapular skinfold thickness; waist and hip circumference; total upper arm and upper arm muscle areas; and BMI
		Byun J.	2020	[28]	Prospective cohort	495	Inverse correlation between severity/type of endometriosis and anthropometric/body composition indicators

Genetic	Gene expression	Rahmioglu N.	2015	[30]	Genome-wide association	10.254	Enrichment of common variants between endometriosis and fat distribution of genetic locus 7p15.2
		Goetz L. G.	2016	[29]	Experimental	30	In a murine model of endometriosis, 4 genes related to weight loss (Mrc1, Rock2, Cyp2r1, and Fabp4) were overexpressed and 2 genes linked to obesity (Igfbp1 and Mmd2) were underexpressed
		**Vilarinho Cardoso J.**	**2017**	31	**Case-control**	**356**	**The polymorphism CYP2C19∗2 is positively associated with both endometriosis and high BMI**
	MicroRNA assays	Zolbin M. M.	2019	[33]	Experimental	20	Endometriosis alters BMI through genetic alterations in adipocyte differentiation and metabolism, propagated by Let-7b and miR-342-3p miRNAs
		**Holdswoth-Carson S. J.**	**2020**	34	**Cross-sectional**	**169**	**No association between BMI and altered endometrial gene expression in women with or without endometriosis**
Molecular	Ghrelin	Dziunycz P.	2009	[52]	Case-control	66	Endometriosis associated with increased peritoneal ghrelin levels
		Milewski L.	2012	[53]	Cross-sectional	20	Colocalization of ghrelin and its receptor in human ovarian endometrioma tissue samples
		Rathore N.	2014	[54]	Cross-sectional	50	Median levels of ghrelin in patients with endometriosis were lower as compared to patients without endometriosis
	Leptin	Matarese G.	2000	[55]	Case-control	28	Early-stage endometriosis is associated with higher leptin levels than advanced-stage disease
		Styer A. K.	2008	[59]	Experimental	12	In a murine model of endometriosis, leptin signaling was necessary for lesion proliferation, early vascular recruitment, and maintenance of neoangiogenesis
		Gungor T.	2009	[58]	Prospective cohort	112	Higher levels of leptin were observed in peritoneal environments of endometriosis subjects
		Choi Y. S.	2013	[61]	Case-control	86	The expressions of leptin and its receptor are induced in ovarian endometrioma tissue samples
		**Shah D. K.**	**2013**	60	**Case-control**	**29.611**	**No statistically significant association between endometriosis and leptin espression**
		Rathore N.	2014	[54]	Cross-sectional	50	Median levels of leptin in patients with endometriosis were higher as compared to patients without endometriosis
		Gonçalves H. F.	2015	[57]	Case-control	25	Serum leptin levels were increased in patients versus controls, whereas there was no difference in peritoneal leptin levels
	Adiponectin	Takemura Y.	2005	[64]	Case-control	80	Adiponectin concentrations in peritoneal fluid of women with endometriosis were significantly lower than those of women without it
		Takemura Y.	2005	[65]	Case-control	78	Adiponectin concenterations in serum of women with endometriosis were significantly lower than those of women without it
		Choi Y. S.	2013	[61]	Case-control	86	The expression of adiponectin and its receptor is similar between endometriosis and normal controls
		**Shah D. K.**	**2013**	60	**Case-control**	**29.611**	**No statistically significant association between endometriosis and adiponectin expression**
	Chemerin	Jin C. H.	2015	[68]	Case-control	79	Chemerin concentrations in peritoneal fluid and serum are higher in women with endometriosis than controls
